# Checkpoint inhibitor induced hepatitis and the relation with liver metastasis and outcome in advanced melanoma patients

**DOI:** 10.1007/s12072-021-10151-4

**Published:** 2021-02-25

**Authors:** Maaike Biewenga, Monique K. van der Kooij, Michel W. J. M. Wouters, Maureen J. B. Aarts, Franchette W. P. J. van den Berkmortel, Jan Willem B. de Groot, Marye J. Boers-Sonderen, Geke A. P. Hospers, Djura Piersma, Rozemarijn S. van Rijn, Karijn P. M. Suijkerbuijk, Albert J. ten Tije, Astrid A. M. van der Veldt, Gerard Vreugdenhil, John B. A. G. Haanen, Alfons J. M. van der Eertwegh, Bart van Hoek, Ellen Kapiteijn

**Affiliations:** 1 Department of Gastroenterology and Hepatology, Leiden University Medical Centre, Leiden, The Netherlands; 2 Department of Medical Oncology, Leiden University Medical Centre, Leiden, The Netherlands; 3 Department of Medical and Surgical Oncology, Netherlands Cancer Institute-Antoni van Leeuwenhoek Hospital, Amsterdam, The Netherlands; 4 Scientific Bureau, Dutch Institute for Clinical Auditing, Leiden, The Netherlands; 5 Department of Medical Oncology, Maastricht University Medical Centre+, Maastricht, The Netherlands; 6 Department of Medical Oncology, Zuyderland Medical Centre, Sittard-Geleen, The Netherlands; 7 Isala Oncology Centre, Isala, Zwolle, The Netherlands; 8 Department of Medical Oncology, Radboud University Medical Centre, Nijmegen, The Netherlands; 9 Department of Medical Oncology, University Medical Centre Groningen, Groningen, The Netherlands; 10 Department of Internal Medicine, Medisch Spectrum Twente, Enschede, The Netherlands; 11 Department of Internal Medicine, Medical Centre Leeuwarden, Leeuwarden, The Netherlands; 12 Cancer Centre, University Medical Centre Utrecht, Utrecht, The Netherlands; 13 Department of Internal Medicine, Amphia Hospital, Breda, The Netherlands; 14 Departments of Medical Oncology and Radiology and Nuclear Medicine, Erasmus Medical Centre Cancer Institute, Rotterdam, The Netherlands; 15 Department of Internal Medicine, Maxima Medical Centre, Eindhoven/Veldhoven, The Netherlands; 16 Department of Medical Oncology, Netherlands Cancer Institute-Antoni van Leeuwenhoek Hospital, Amsterdam, The Netherlands; 17 Department of Medical Oncology, Cancer Centre Amsterdam, Amsterdam UMC, Vrije Universiteit Amsterdam, Amsterdam, The Netherlands

**Keywords:** Ipilimumab, Nivolumab, CTLA-4 inhibitor, PD-1 inhibitor, Drug-induced Hepatitis, Risk factors, Progression-Free Survival, Overall survival, Immune-related adverse events, Liver metastasis

## Abstract

**Background:**

Checkpoint inhibitor-induced hepatitis is an immune-related adverse event of programmed cell death protein 1 (PD-1) inhibition, cytotoxic T-lymphocyte associated 4 (CTLA-4) inhibition or the combination of both. Aim of this study was to assess whether checkpoint inhibitor-induced hepatitis is related to liver metastasis and outcome in a real-world nationwide cohort.

**Methods:**

Data from the prospective nationwide Dutch Melanoma Treatment Registry (DMTR) was used to analyze incidence, risk factors of checkpoint inhibitor-induced grade 3–4 hepatitis and outcome.

**Results:**

2561 advanced cutaneous melanoma patients received 3111 treatments with checkpoint inhibitors between May 2012 and January 2019. Severe hepatitis occurred in 30/1620 (1.8%) patients treated with PD-1 inhibitors, in 29/1105 (2.6%) patients treated with ipilimumab and in 80/386 (20.7%) patients treated with combination therapy. Patients with hepatitis had a similar prevalence of liver metastasis compared to patients without hepatitis (32% vs. 27%; *p* = 0.58 for PD-1 inhibitors; 42% vs. 29%; *p* = 0.16 for ipilimumab; 38% vs. 43%; *p* = 0.50 for combination therapy). There was no difference in median progression free and overall survival between patients with and without hepatitis (6.0 months vs. 5.4 months progression-free survival; *p* = 0.61; 17.0 vs. 16.2 months overall survival; *p* = 0.44).

**Conclusion:**

Incidence of hepatitis in a real-world cohort is 1.8% for PD-1 inhibitor, 2.6% for ipilimumab and 20.7% for combination therapy. Checkpoint inhibitor-induced hepatitis had no relation with liver metastasis and had no negative effect on the outcome.

**Supplementary Information:**

The online version contains supplementary material available at 10.1007/s12072-021-10151-4.

## Introduction

The introduction of immune checkpoint inhibitors has significantly improved the 5-year survival of patients with advanced melanoma [1]. The checkpoint inhibitors registered for the treatment of advanced melanoma are the programmed cell death protein 1 (PD-1) inhibitors nivolumab and pembrolizumab, the cytotoxic T-lymphocyte associated 4 (CTLA-4) inhibitor ipilimumab and the combination of ipilimumab and nivolumab. The use of immune checkpoint inhibitors can lead to an array of immune-related adverse events (IRAE), including checkpoint inhibitor-induced hepatitis.

Based on data from clinical trials, the incidence of grade 3–4 checkpoint inhibitor-induced hepatitis is 1–17%, depending on the checkpoint inhibitor used [2–9]. For ipilimumab the reported incidences are between 2–9% [3, 4, 8], for PD-1 inhibitors incidences between 1 and 4% have been reported [7, 8] and for combination therapy the incidence varies between 8–17% [6, 8, 9]. Only small studies have described characteristics of checkpoint inhibitor-induced hepatitis and no risk factors have been reported [2, 10].

Severity of hepatitis can be graded based on the alanine aminotransferase (ALT) and aspartate aminotransferase (AST) level. Treatment can be continued with grade 1 (ALT or AST 1–3 times elevated). With grade 2 (ALT or AST 3–5 times elevated) the next cycle should be delayed and prednisolone started if transaminases continue to rise. Grade 3 (ALT or AST 5–20 times elevated) and grade 4 (ALT or AST > 20 times elevated) should be treated with 1–2 mg/kg prednisolone per day according to guidelines [11, 12]. If the response is insufficient within 2–3 days, mycophenolate mofetil and/or tacrolimus can be added. Typically, checkpoint inhibitor-induced hepatitis responds well to treatment with corticosteroids although tapering of prednisolone can take 6–8 weeks [13].

Incidence of IRAEs differs between different tumors types and can be correlated to treatment response. Development of vitiligo is much more frequent in melanoma patients than in patients with other solid tumors [14–16]. Melanoma patients with vitiligo have a better overall survival compared to patients without vitiligo [17]. Lung cancer patients have a higher risk of developing pneumonitis as IRAE [18]. However, this did not seem to influence the progression-free or overall survival [19]. Based on these findings, we hypothesized that the presence of liver metastasis could be a possible risk factor for checkpoint inhibitor-induced hepatitis.

A correlation between the occurrence of hepatitis and outcome is currently unknown. Stopping of immune checkpoint inhibitor therapy and the start of immunosuppressive treatment might decrease survival. Patients with liver metastasis have a lower rate of response compared to patients with visceral disease not involving the liver [20]. All these factors could influence survival in patients with hepatitis.

The aim of this study was to assess if checkpoint inhibitor-induced hepatitis is related to liver metastasis and outcome. The secondary aim was to assess the incidence and current treatment of patients with checkpoint inhibitor-induced grade 3–4 hepatitis in a unique real-world, nationwide prospective registry.

## Methods

All advanced melanoma patients in the Netherlands are registered in the Dutch Melanoma Treatment Registry (DMTR). To ensure safety and quality of care, all data regarding the type of melanoma, given treatment, incidence of grade 3–4 adverse events and treatment outcome are registered prospectively [21]. Treatment with immune checkpoint inhibition including PD-1 inhibitors (nivolumab or pembrolizumab), CTLA-4 inhibitor ipilimumab and combination therapy with nivolumab and ipilimumab or targeted therapy with serine/threonine-protein kinase B-Raf (BRAF) inhibitors (vemurafenib, dabrafenib and encorafenib) and mitogen-activated protein kinase enzymes (MEK) inhibitors (trametinib, cobimetinib and binimetinib) are registered in treatment episodes. A treatment episode starts when a treatment is started and ends when a patient dies or a different treatment is initiated. If patients are switched to a different therapy, multiple treatment episodes are available for these patients. This registry has a nationwide coverage because registration is mandatory for reimbursement and all systemic therapy for melanoma is given in the 14 designated melanoma treatment centers. Data from July 2012 until July 2013 were retrospectively entered. From July 2013 until January 2019 data is prospectively entered by trained data managers and checked by treating physicians. In compliance with Dutch regulations, the medical ethical committee of the Leiden University Medical Centre judged that the DMTR was not subject to the Medical Research Involving Human Subjects Act.

All cutaneous melanoma patients registered in the DMTR database who received at least one cycle with PD-1 inhibitor, ipilimumab or combination therapy of nivolumab and ipilimumab between May 2012 and January 2019 were eligible for inclusion. Patients with missing liver toxicity data, a follow-up of less than 4 weeks and patients with uveal melanoma were excluded.

The different treatment regimens (PD-1 inhibitor, ipilimumab and combination therapy) were analysed separately. Treatment was administered in 2-week cycles for nivolumab and 3-week cycles for pembrolizumab, ipilimumab and combination therapy. Ipilimumab treatment was stopped after four cycles. Patients with combination therapy continued with nivolumab after four cycles of combination therapy. If patients were treated with different checkpoint inhibitors in different treatment episodes, all treatment episodes were included for analysis. If patients were treated in multiple episodes with the same checkpoint inhibitor, only the first treatment episode was included. Previous treatment episodes with targeted or immunotherapy were analysed as a risk factor for hepatitis.

Checkpoint inhibitor-induced hepatitis was defined as grade 3 (ALT or AST 5–20 times the upper limit of normal) or grade 4 (ALT or AST > 20 times the upper limit of normal) according to the CTCAE, version 4.0. Lactate dehydrogenase (LDH) was measured before the start of treatment with an immune checkpoint inhibitor. Elevated LDH was defined as LDH > 250 U/L. Presence of liver metastasis was based on a CT scan or a fluorodeoxyglucose (FDG) PET CT scan performed within 3 months of the start of treatment.

### Statistics

All data are presented as mean (standard deviation) unless specified otherwise. Mann–Whitney *U* test was used to test for significance for continuous variables. Chi-square test was used for categorical variables. Univariate logistic regression was used for determining risk factors for the development of hepatitis. For survival analysis, Kaplan Meier curves, log-rank test and univariate and multivariate Cox regression analysis were used where appropriate. In multivariate analysis presence of immune checkpoint inhibitor-induced hepatitis was corrected for known risk factors including age, liver metastasis, cerebral metastasis, LDH, > 3 organs affected, WHO status and checkpoint inhibitor regimen. *p* value < 0.05 was considered significant. IBM SPSS Statistics for Windows, version 25 was used for statistical analysis.

## Results

**Fig. 1:**
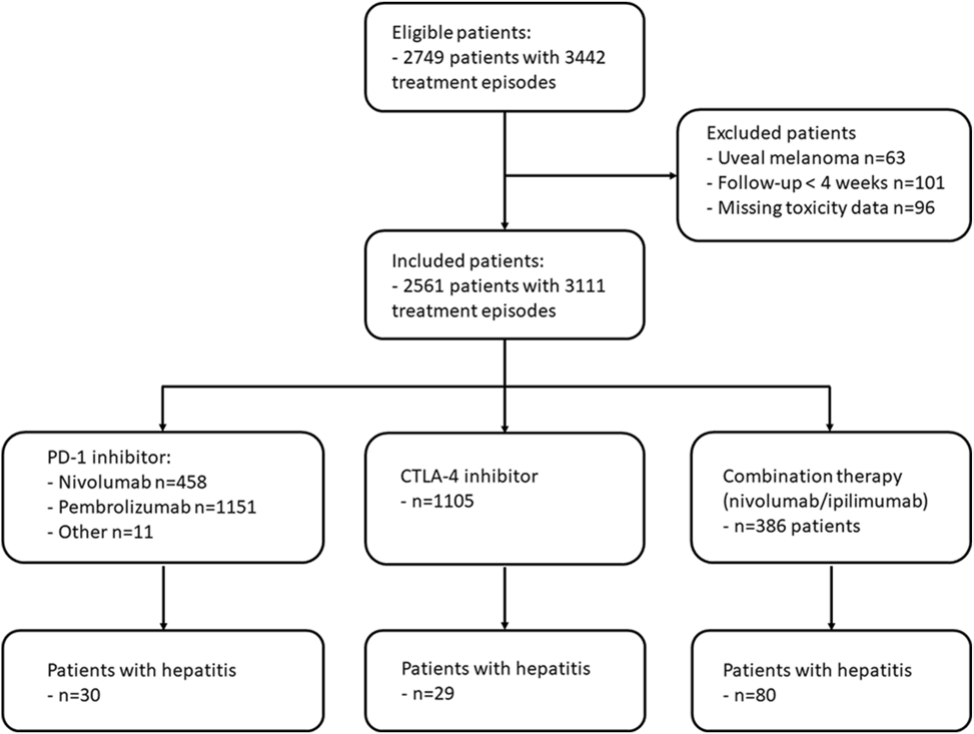
Flowchart of eligible and included patients and treatment episodes

2749 patients were treated with checkpoint inhibitors for advanced melanoma in the Netherlands between May 2012 and January 2019. In total 2561 patients with 3111 treatment episodes met the inclusion criteria. Treatment with PD-1 inhibitors was given in 1620 patients, ipilimumab in 1105 patients and combination therapy in 386 patients (Fig. 1). Multiple treatment episodes were registered in 550 patients.

Checkpoint inhibitor-induced hepatitis occurred in 30 (1.8%) patients treated with PD-1 inhibition, 29 (2.6%) patients treated with ipilimumab and in 80 (20.7%) patients treated with combination therapy. Patients treated with combination therapy had a higher risk of developing hepatitis compared to monotherapy (HR 10.66; 95% CI 7.44–15.28; *p* < 0.001). One patient had an episode of hepatitis after the use of combination therapy and another episode of hepatitis when PD-1 monotherapy was restarted. Only two patients (1.4%) with hepatitis were known with pre-existing liver disease before the start of immunotherapy. Additional information on the liver disease was not registered.

### Previous therapies

In 1048 (65%) of the 1620 patients treated with PD-1 inhibition, PD-1 inhibition was the first-line treatment. The remaining 572 patients had been treated in the previous treatment episode with ipilimumab in 239 patients (15%), combination therapy in 24 patients (1%), and BRAF or BRAF/MEK inhibitors in 309 patients (19%).

In 738 (67%) of the 1105 patients treated with ipilimumab, ipilimumab was the first-line treatment. The remaining 367 patients had been treated in the previous treatment episode with PD-1 inhibition in 118 patients (11%), combination therapy in 2 patients (0.2%) and BRAF or BRAF/MEK inhibitors in 260 patients (24%).

In 219 (57%) of the 386 patients treated with combination therapy, combination therapy was the first-line treatment. The remaining 167 patients had been treated in the previous treatment episode with PD-1 inhibition in 10 patients (2.6%) and BRAF or BRAF/MEK inhibitors in 157 (41%) patients.

### Risk factors for PD-1 inhibitor-induced hepatitis

Age and gender were similar between patients with PD-1 inhibitor-induced hepatitis and patients with PD-1 inhibitor treatment without hepatitis (Table 1). Liver metastases were present in 32% of the patients with hepatitis and 27% of the patients without hepatitis (*p* = 0.583). Patients with hepatitis more often had an elevated LDH before treatment compared to patients without hepatitis (52% vs. 29%; *p* = 0.009). No difference was found in WHO status, number of organs affected or the previous type of treatment (Table 1).

**Table 1:** Baseline characteristics of patients treated with PD-1 inhibitor with and without the development of ≥ grade 3 hepatitis

	PD-1 inhibitor		*p* value
	Hepatitis	No hepatitis	
*N*	30	1590	
Age in years	64.8	63.2	0.62
Female gender	12 (40%)	653 (41%)	0.91
WHO status			0.33
0	13 (48%)	841 (58%)	
1–3	14 (52%)	622 (42%)	
LDH			0.009
Normal	14 (48%)	1096 (71%)	
> 250	15 (52%)	457 (29%)	
Organs affected			0.68
< 3	13 (48%)	703 (52%)	
≥ 3	14 (52%)	646 (48%)	
Treatment history			
Immunotherapy	6 (20%)	336 (21%)	0.88
Ipilimumab	4 (13%)	305 (19%)	
Combination therapy	2 (7%)	31 (2%)	
Targeted therapy	6 (20%)	347 (22%)	0.81
BRAF	1 (3%)	157 (10%)	
BRAF/MEK	5 (17%)	190 (12%)	
Location metastasis			
Liver	9 (32%)	404 (27%)	0.58
Lung	11 (38%)	794 (54%)	0.083
Cerebral	8 (28%)	395 (27%)	0.96
Gastrointestinal	5 (17%)	117 (8%)	0.070
Bone	4 (14%)	372 (25%)	0.16
Lymph nodes	20 (71%)	785 (53%)	0.053
Skin	10 (35%)	477 (32%)	0.81
Other	10 (36%)	545 (37%)	0.90

*PD-1* programmed cell death protein 1, *WHO* world health organization, *LDH* lactate dehydrogenase, *BRAF* serine/threonine-protein kinase B-Raf, *MEK* mitogen-activated protein kinase enzymes

### Risk factors for ipilimumab induced hepatitis

There were no differences in age and gender between patients with ipilimumab-induced hepatitis and ipilimumab-treated patients without hepatitis (Table 2). Liver metastases were present in 42% of the patients with hepatitis compared to 29% of the patients without hepatitis (*p* = 0.16). Previous treatment with immunotherapy was more prevalent in patients with hepatitis compared to patients without hepatitis (31% vs. 13%; *p* = 0.004). No differences were found in WHO status, LDH, number of organs affected or previous treatment with targeted therapy (Table 2).

**Table 2:** Baseline characteristics of patients treated with ipilimumab with and without the development of ≥ grade 3 hepatitis

	Ipilimumab		*p* value
	Hepatitis	No hepatitis	
*N*	29	1076	
Age in years	58.9	60.3	0.92
Female gender	9 (31%)	446 (41%)	0.26
WHO status			0.26
0	20 (74%)	613 (64%)	
1–3	7 (26%)	352 (36%)	
LDH			0.57
Normal	23 (79%)	779 (75%)	
> 250	6 (21%)	264 (25%)	
Organs affected			0.58
< 3	14 (56%)	480 (50%)	
≥ 3	11 (44%)	474 (50%)	
Treatment history			
Immunotherapy	9 (31%)	136 (13%)	0.004
PD-1	9 (31%)	134 (12%)	
Combination therapy	0 (0%)	2 (0.2%)	
Targeted therapy	5 (17%)	255 (24%)	0.42
BRAF	4 (14%)	190 (18%)	
BRAF/MEK	1 (3%)	65 (6%)	
Location metastasis			
Liver	11 (42%)	301 (29%)	0.16
Lung	13 (50%)	571 (56%)	0.55
Cerebral	3 (12%)	241 (24%)	0.17
Gastrointestinal	4 (15%)	73 (7%)	0.11
Bone	5 (19%)	259 (25%)	0.48
Lymph nodes	11 (42%)	587 (57%)	0.13
Skin	10 (39%)	350 (34%)	0.65
Other	11 (42%)	424 (41%)	0.90

*WHO* world health organization, *LDH* lactate dehydrogenase, *PD-1* programmed cell death protein 1, *BRAF* serine/threonine-protein kinase B-Raf, *MEK* mitogen-activated protein kinase enzymes

### Risk factors for combination therapy induced hepatitis

Patients with combination therapy-induced hepatitis were younger than combination therapy treated patients without hepatitis (53.2 years vs. 56.9 years; *p* = 0.02). Prevalence of liver metastases was similar in patients with and without hepatitis (38% vs. 43%; *p* = 0.50). Patients with hepatitis less often had an elevated LDH before treatment compared to patients without hepatitis (39% vs. 54%; *p* = 0.023). No difference was found in gender, WHO status, number of organs affected or previous treatment (Table 3).

**Table 3:** Baseline characteristics of patients treated with combination therapy (ipilimumab/nivolumab) with and without the development of ≥ grade 3 hepatitis

	Combination therapy		*p* value
	Hepatitis	No hepatitis	
*N*	80	306	
Age in years	53.2	56.9	0.010
Female gender	31 (39%)	124 (41%)	0.77
WHO status			0.69
0	38 (51%)	141 (49%)	
1–3	36 (49%)	148 (51%)	
LDH			0.023
Normal	47 (61%)	137 (46%)	
> 250	30 (39%)	158 (54%)	
Organs affected			0.31
< 3	27 (41%)	92 (34%)	
≥ 3	39 (59%)	177 (66%)	
Treatment history			
Immunotherapy	4 (5%)	31 (10%)	0.16
PD-1	4 (5%)	30 (10%)	
Ipilimumab	0 (0%)	1 (0.3%)	
Targeted therapy	27 (34%)	130 (43%)	0.16
BRAF	0 (0%)	2 (1%)	
BRAF/MEK	27 (34%)	128 (42%)	
Location metastasis			
Liver	29 (38%)	124 (43%)	0.50
Lung	43 (57%)	172 (59%)	0.67
Cerebral	30 (41%)	143 (50%)	0.19
Gastrointestinal	5 (6.8%)	32 (11%)	0.28
Bone	21 (28%)	120 (41%)	0.049
Lymph nodes	40 (54%)	159 (55%)	0.93
Skin	21 (27%)	82 (28%)	0.95
Other	32 (43%)	124 (42%)	0.89

*WHO* world health organization, *LDH* lactate dehydrogenase, *PD-1* programmed cell death protein 1, *BRAF* serine/threonine-protein kinase B-Raf, *MEK* mitogen-activated protein kinase enzymes

### Additional immune-related adverse events

Additional IRAEs were present in 3 (10%) patients with PD-1 inhibitor-induced hepatitis, 10 (34%) patients with ipilimumab induced hepatitis and 29 (36%) patients with combination therapy-induced hepatitis.

**Fig. 2:**
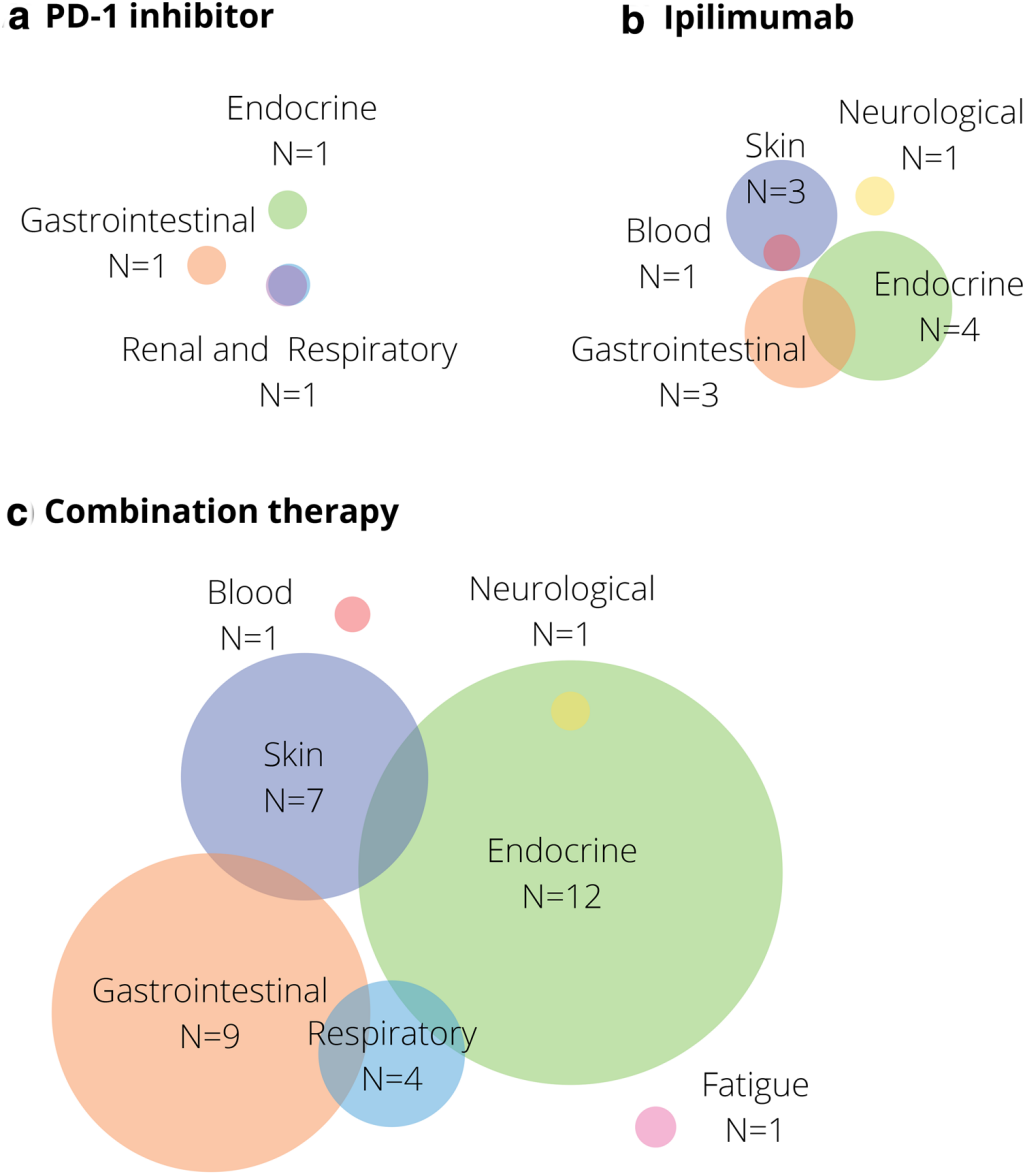
Additional immune related adverse events in patients with **a** PD-1 inhibitor induced hepatitis, **b** ipilimumab induced hepatitis and **c** combination therapy induced hepatitis. Overlapping circles represent patients with more than 1 additional immune-related adverse event

Additional IRAEs consisted mainly of endocrine toxicity in 17 patients, gastrointestinal toxicity in 13 patients and skin toxicity in 10 patients. In 10 patients, two additional IRAEs were present. The distribution of additional IRAEs in patients with hepatitis is presented in Fig. 2.

### Treatment of immune checkpoint inhibitor-induced hepatitis

Hepatitis occurred after median 12 weeks of PD-1 inhibitor treatment (range 1–98 weeks), 6 weeks of ipilimumab treatment (range 1–16 weeks) and 6 weeks of combination therapy (range 1–13 weeks). In 12 patients (41%) hepatitis occurred 1–7 weeks after the fourth and final treatment cycle of ipilimumab. In 2 patients (2.5%) combination therapy-induced hepatitis occurred during the maintenance phase with nivolumab.

For 15 episodes treatment details were not registered. Treatment with corticosteroids was started in 123 of the 124 (99%) episodes of checkpoint inhibitor-induced hepatitis. One patient with ipilimumab induced hepatitis who was not treated with corticosteroids or immunosuppressants died 4 months after the occurrence of hepatitis due to melanoma progression. In 25 (20%) treatment episodes, second-line immunosuppressive therapy was given in addition to corticosteroids. The type of second-line immunosuppressive therapy was not registered. Second-line immunosuppressive therapy was given in 9% of the patients with PD-1 inhibitor-induced hepatitis, in 20% of the patients with ipilimumab induced hepatitis and in 24% of the patients with combination therapy induced hepatitis (*p* = 0.29). Need for second-line immunosuppressive therapy was similar between patients with and without liver metastasis (23% vs. 21%; *p* = 0.76).

Approximately 35% of the patients with hepatitis were admitted to the hospital (PD-1 inhibitors 37%, ipilimumab 41% and combination therapy 35%). In admitted patients, additional IRAEs were registered in 36% of PD-1 induced hepatitis, 42% in ipilimumab induced hepatitis and 64% of combination therapy induced hepatitis. Three patients, one in each treatment regimen, died due to the toxicity of which 2 patients had PD-1 inhibitor-induced colitis and ipilimumab induced nephritis as additional IRAEs.

After hepatitis resolved, a different immunotherapy or targeted therapy was started in 9 patients with PD-1 inhibitor-induced hepatitis and 12 patients with ipilimumab- induced hepatitis. None of these patients developed another episode of hepatitis. In 42 patients (53%) with combination therapy induced hepatitis, maintenance treatment with PD-1 inhibitors was (re)started after the hepatitis was resolved. One patient developed another episode of hepatitis after the start of PD-1 inhibition.

### Progression-free and overall survival

Median follow-up time was 6.5 months. Median progression-free survival and median overall survival did not differ between patients with and without hepatitis (6.0 months and 5.4 months progression-free survival; *p* = 0.61; 17.0 and 16.2 months overall survival; *p* = 0.44). When corrected in multivariate analysis, association between checkpoint inhibitor-induced hepatitis and progression-free survival and overall survival remained non-significant (progression-free survival: HR 0.88 (95% CI 0.69–1.11) *p* = 0.21; overall survival HR 0.79 (95% CI 0.58–1.06); *p* = 0.11; Table 4). Results of univariate analysis can be found in supplementary Table 1.

**Table 4:** Multivariate analysis of progression-free and overall survival

	Hazard Ratio (95% CI)	*p* value
Progression free survival		
Hepatitis	0.88 (0.69–1.11)	0.27
Liver metastasis	1.32 (1.18–1.48)	< 0.001
Cerebral metastasis	1.30 (1.16–1.46)	< 0.001
Age	0.99 (0.99–1.00)	0.39
> 3 organs affected	1.18 (1.05–1.31)	0.004
Elevated LDH	1.39 (1.24–1.55)	< 0.001
WHO		
1	1.25 (1.13–1.40)	< 0.001
2–3	1.75 (1.41–2.16)	< 0.001
Type checkpoint inhibitor		
Ipilimumab	Reference	
PD-1	0.52 (0.47–0.58)	< 0.001
Combination therapy	0.59 (0.50–0.70)	< 0.001
Overall survival		
Hepatitis	0.79 (0.58–1.06)	0.11
Liver metastasis	1.49 (1.31–1.71)	< 0.001
Cerebral metastasis	1.55 (1.35–1.78)	< 0.001
Age	1.01 (1.00–1.01)	0.001
> 3 organs affected	1.18 (1.04–1.35)	0.014
Elevated LDH	1.74 (1.53–1.98)	< 0.001
WHO		
1	1.41 (1.25–1.60)	< 0.001
2–3	2.17 (1.71–2.75)	< 0.001
Type checkpoint inhibitor		
Ipilimumab	Reference	
PD-1	0.65 (0.57–0.74)	< 0.001
Combination therapy	0.74 (0.60–0.91)	0.005

*CI* confidence interval, *LDH* lactate dehydrogenase, *WHO* world health organization, *PD-1* programmed cell death protein 1

## Discussion

In the largest cohort reported thus far, we observed no relation between checkpoint inhibitor-induced hepatitis and the presence of liver metastasis. The incidences of PD-1 inhibitor and ipilimumab induced grade 3–4 hepatitis were 1.8% and 2.6%, respectively, which is comparable to previously published incidences [3, 4, 7, 8]. The incidence of combination therapy-induced hepatitis was 20.7%, which is higher than previously published frequencies of 9–17% [6, 8, 9].

The use of immune checkpoint inhibitor therapy is rapidly increasing as these drugs will be approved for more indications. Immune checkpoint inhibition has currently been approved in the Netherlands for advanced stages of melanoma, lung cancer, renal cell carcinoma, bladder carcinoma, squamous cell carcinoma of head and neck and Hodgkin lymphoma [1, 22–26]. Furthermore, immunotherapy with checkpoint inhibitors has recently been approved as adjuvant treatment in stage III melanoma and is under investigation in many tumor types as adjuvant and neoadjuvant treatment [27, 28].

As an increase in checkpoint inhibitor-induced hepatitis can be expected, it is important to identify risk factors. Presence of liver metastasis was not a risk factor for hepatitis in patients treated with checkpoint inhibitors. Similarly, in patients with hepatocellular carcinoma the incidence of grade ≥ 3 hepatitis was found to be 2% for PD-1 inhibitors and 20% for combination therapy [29]. These frequencies are comparable to our findings and also argue against an association between an affected liver and immune checkpoint inhibitor-induced hepatitis.

For PD-1 inhibitor-induced hepatitis, elevated LDH before treatment was related to an increased risk. A clear explanation of this finding is difficult. Elevated LDH is related to total tumor load and is highly prognostic for a poor progression-free and overall survival. In addition, LDH is an independent negative predictor for therapy response in PD-1 inhibitor and ipilimumab monotherapy [30, 31]. More research is needed to clarify the relation between LDH and PD-1 inhibitor-induced hepatitis.

For ipilimumab-induced hepatitis, previous treatment with immunotherapy, mainly PD-1 inhibitor monotherapy, increased the risk of hepatitis. The effect of PD-1 inhibition might partially continue after stopping the treatment. Treatment with ipilimumab after PD-1 inhibition could then result in combined inhibition of PD-1 and CTLA-4 and increase the risk of hepatitis.

For combination therapy-induced hepatitis, younger age was a risk factor in the current study. Younger patients may have a more active immune system which can lead to hepatitis or other IRAEs [32]. A recent study showed no increased rate of grade 3–4 side effects in general in younger patients compared to older adults [33]. However, for combination therapy-induced hepatitis it does seem a relevant risk factor.

Checkpoint inhibitor-induced hepatitis occurred after median 6 weeks in ipilimumab and combination therapy and after median 12 weeks for PD-1 inhibitors. This is comparable to the previously reported 6–7 weeks in ipilimumab and combination therapy and 14 weeks in PD-1 inhibitors [11, 34]. As the ranges are wide, physicians should be aware that hepatitis can still occur in the later stages of treatment and even after treatment has stopped. Checkpoint inhibitor-induced hepatitis was treated with corticosteroids in all patients except one. Second-line immunosuppression was necessary in 20% of the patients. The type of second-line therapy was not registered, but we can assume that this was mostly mycophenolate mofetil and/or tacrolimus since these drugs are added to corticosteroid treatment in case of insufficient response according to the ESMO guidelines [11]. The rate of 20% need for second-line immunosuppressive treatment is lower than the 45% (9 of 20 patients) previously reported [10].

If a new targeted or immunotherapy was started after hepatitis was resolved, recurrence of hepatitis was rare. The decision to start a new treatment is dependent on many factors including the antitumor response.

IRAEs are a sign of activated immunity which may promote an antitumor response. A recent meta-analysis showed a better survival for patients with IRAEs compared to patients without IRAEs [35]. Immunosuppressive therapy, especially anti-TNF therapy, may be associated with decreased overall survival [36]. Although treatment with immune checkpoint inhibitors was stopped earlier due to hepatitis and immunosuppressive treatment was given, checkpoint inhibitor-induced hepatitis had no negative or positive effect on progression-free survival and overall survival.

A strength of this study is the prospective nationwide coverage of all patients with advanced melanoma who were treated with immune checkpoint inhibitors. This resulted in the largest cohort of checkpoint inhibitor-induced hepatitis to our knowledge. Due to nationwide registration a reliable estimate of incidences, current treatment of checkpoint inhibitor-induced hepatitis and outcomes can be given in a real-world cohort. In addition, the size of the cohort allowed us to analyze the different types of checkpoint inhibitor-induced hepatitis separately, which has not been possible in other smaller studies up till now. A weakness of the study was that some liver-specific variables such as AST, ALT, alkaline phosphatase (AP) and gamma-glutamyltransferase (GGT), liver biopsies results and type of second-line immunosuppression were not registered.

Our study shows that the incidence of grade 3–4 hepatitis in a real-world cohort is 1.7% for PD-1 inhibitor treatment, 2.6% for ipilimumab treatment and 20.7% for combination therapy. Hepatitis was not related to liver metastasis and had no negative effect on survival. As a rise in the number of patients with checkpoint inhibitor-induced hepatitis can be expected, it is important that oncologists and hepatologists have knowledge of this IRAE. Further prospective research and evidence-based treatment guidelines are important to optimize treatment strategies for checkpoint inhibitor-induced hepatitis.

## Supplementary Information

Below is the link to the electronic supplementary material.

Supplementary file1 (DOCX 14 KB)

## Data Availability

The data that support the findings of this study are available on reasonable request from the corresponding author. The data are not publicly available due to privacy and data legislation.

## Abbreviations

PD-1: Programmed cell death protein 1
CTLA-4: Cytotoxic T-lymphocyte associated 4
IRAE: Immune-related adverse event
ALT: Alanine aminotransferase
AST: Aspartate aminotransferase
DMTR: Dutch Melanoma Treatment Registry
BRAF: Serine/threonine-protein kinase B-Raf
MEK: Mitogen-activated protein kinase enzymes
LDH: Lactate dehydrogenase
HR: Hazard ratio
AP: Alkaline phosphatase
GGT: Gamma-glutamyltransferase
