# Supplementary material for: Checkpoint inhibitor induced hepatitis and the relation with liver metastasis and outcome in advanced melanoma patients
Source: Hepatol Int. 2021 Feb 25;15(2):510–9. doi: 10.1007/s12072-021-10151-4 (PMC8144142; doi:10.1007/s12072-021-10151-4)
Supplement: Supplementary file 1 — Supplementary file1 (DOCX 14 KB) [file 12072_2021_10151_MOESM1_ESM.docx]

**Supporting information**

Supplementary table 1: results of univariate Cox regression analysis for progression free and overall survival

|  | Progression free survival | | Overall survival | |
| --- | --- | --- | --- | --- |
|  | Hazard ratio | P-value | Hazard ratio | P-value |
| Age | 0.99 (0.99-1.00) | 0.026 | 1.00 (0.99-1.01) | 0.11 |
| Hepatitis | 0.95 (0.78-1.16) | 0.61 | 0.91 (0.70-1.17) | 0.44 |
| Liver metastasis | 1.41 (1.28-1.56) | <0.001 | 1.65 (1.48-1.84) | <0.001 |
| Cerebral metastasis | 1.40 (1.27-1.55) | <0.001 | 1.68 (1.50-1.89) | <0.001 |
| >3 organs affected | 1.42 (1.29-1.56) | <0.001 | 1.63 (1.46-1.82) | <0.001 |
| Elevated LDH | 1.45 (1.32-1.59) | <0.001 | 1.90 (1.71-2.12) | <0.001 |
| WHO status  0  1  2-3 | Reference  1.32 (1.20-1.46)  1.70 (1.40-2.06) | <0.001  <0.001 | Reference  1.64 (1.46-1.84)  2.49 (2.00-3.08) | <0.001  <0.001 |
| Type checkpoint inhibitor  Ipilimumab  PD-1  Combination therapy | Reference  0.55 (0.50-0.61)  0.69 (0.60-0.80) | <0.001  <0.001 | Reference  0.72 (0.66-0.81)  0.96 (0.80-1.14) | <0.001  0.61 |
